# Nitrous Oxide Experiments

**Published:** 1873-08

**Authors:** S. P. Cutler


### ARTICLE II.

### *Nitrous Oxide Experiments.*

Continued from May Number, 1872.

BY S. P. CUTLER, M. D., D. D. S.

Prof. Barker, in his work on Nitrous Oxide, page 50, says: "My opinion is, that the chief danger in nitrous oxide consists in the rapid formation of carbonic acid gas, which is due to the excessive disintegration and waste that continue while the system is under its influences." Same page, says: "If a mouse is placed under a receiver, and allowed to breathe nothing but nitrous oxide, it will die, and on opening the heart and blood vessels, the blood will be found to present a dark color, usually present when death results from asphyxia."

Page 55, author says: "The danger of nitrous oxide consists in the fact that it increases oxidation of the fluids and solids of the body, and also acts as a stimulant to the brain and nervous system; where there exists any predisposition to congestion or inflammation, the administration of nitrous oxide may develop this latent tendency, and a fatal result ensue."

On the contrary, my experiments fully demonstrate the fact that the gas retards oxidation throughout the body, rather than increasing it; which fact will be made more manifest further on. The gas produces symptoms closely allied to catalepsy if not identical; that disease though not dangerous, is not pleasant to witness. The same might be said of the gas. The above writer, says: "The gas increases oxidation of the fluids and solids of the body, and acts as a stimulant to the brain and nervous system." When every symptom speaks to the contrary, as in cases of catalepsy, a want of oxidation is everywhere manifest.

In a pamphlet on nitrous oxide, by S. R. Divine, M. D., page 23, says: "Nitrous oxide supports respiration, and exalts the functions of the animal system. It is composed of the same elements that constitute the air we breathe, and hence must be most nearly normal. It produces *carbonic*

*acid* by decomposition in the circulation; but this is not a foreign substance, for the air generates it there also." Same page, says: "I will venture a theory as to the primary cause of anæsthesia. I have supposed it to be the introduction into the blood of a foreign substance (or in the case of nitrous oxide, carbonic acid, beyond the normal quantity.) Possibly it is essential that the substance be carbonaceous, as is the case directly or indirectly, with all known anæsthetics. I am of opinion that nitrous oxide owes its anæsthetic effect to the formation of carbonic acid in the circulation faster than it is eliminated.

Page 19, says: "*Expired air* contains about on an average, three and a half per cent. of carbonic acid by measure. Twenty per cent. of carbonic acid in air, renders it destructive to life. Such a mixture will first cause drowsiness, then total loss of muscular power, afterwards insensibility, coma and death. There can be no doubt that in some cases where a small quantity of gas has been used, that the patients have been asphyxiated with carbonic acid, instead of *anæsthetized* with nitrous oxide. The quantity of *nitrous oxide* that is converted into carbonic acid in the blood, is probably quite small, but still the expirations contain a larger amount of carbonic acid than when air is used."

Do we know precisely the functions gas performs in its rounds of the circulation? Does this gas enter into the red corpuscles? If so, by what law of affinity does it enter them, and by what law leaves them again, either in the systemic capillaries or lungs on the return currents. Who shall answer this important question?

The same might be asked of chloroform and all other anæsthetic vapors. None of these vapors perform vital functions; if so, anæsthesia could not be superinduced; their functions in the economy must be regarded as anti-vital in all respects; a retrograde action. They replace simple air, take the place of it, and arrest oxidation throughout the organism in proportion to the extent it is carried.

Any innocuous vapor, as nitrogen or hydrogen, might be used with somewhat similar results; in fact the gases have been inhaled with impunity. Do we know whether or not any oxidation takes place with the oxygen that is already in the system when the gas or any other vapor is used after the blood becomes saturated with it?

We have good reasons to believe the negative of this question, from all the symptoms produced, such as loss of sensation, muscular power and consciousness, blueness of skin lips, difficult respiration, cadaverous expression, and all other attendant phenomena.

None of these are normal signs; none of these are healthful signs; on the contrary morbid indications—signs none of us like to witness; they are startling, and awaken fears in the most courageous *gastodian.*

When an individual is under asphyxia he is insensible and unconscious of pain, from the arrestation of a chief vital function; that of atmospherical or normal respiration, coma and syncope, which are the effects of the arrestation of the other two vital functions, or their suspension, also renders the person unconscious to pain from traumatic injuries. When oxidation in the blood and other tissues becomes arrested from any cause, nervous transmission to and from the brain become in proportion arrested, more especially to the brain, which carries sensation. It seems that these nervous currents, the result of oxidation, simultaneously in every cell of the entire body, are indispensable to the conveyance of pain from any point to the brain.

From *a priori* conclusion, we infer that transmission becomes arrested, or if actually taking place, there is no consciousness or mental recognition. Volition, also, and consciousness succumbs in proportion to the extent it is carried. The same would equally apply to both syncope and coma. Sensibility, volition and consciousness, are in exact proportion to the extent of the effect attained. Again, when vital air is excluded, and something else substituted in its stead, there must necessarily be a retrogradence of vital functions, *paripassu* taking place, co-extensive with the time and completeness of effect. Cause and effect have to go hand in hand; as the cause becomes more absolute, so also does the effect become more absolute.

Richardson says, of his ether spray producing local anæsthesia, "that sensation is cut off from the brain, from the point chilled, by virtue of the cold arresting nervous transmission." This is simply the effect, the true cause being the suspension of oxidation of the chilled point, and in consequence, no nerve forces being generated at such point, no transmission takes place. Any intermediate available point where an operation is to be performed, sprayed and chilled over the track of an afferent nerve, provided all circuitous routes could be guarded, we would also prevent the function of pain. But such cases are rare and impracticable.

All oxidation ceases at the freezing point, either in or out of the body, and gradually diminishes as that point is reached; hence, a person with a frost bit finger could suffer amputation without pain, as there is neither oxidation or transmission. Wherever there is pain felt, there is undue oxidation either from disease or injury of a part taking place. This undue oxidation causes undue heat, the normal amount of magnetic force is not evolved, as the injured or weakened part gives oxidation an undue preponderence over nutrition. An injured or weakened part generates too much heat, and too little nervous magnetic force, as the one is rapidly converted into the other under certain conditions. Oxidation causes molecular disturbance, *i. e.*, motion among molecules. This motion in one case means heat, in the other, magnetism: motion being the resultant not the cause *per se*.

From second report of the Odontological Society of Great Britain, and the committee of management of the Dental Hospital of London, to inquire into the value and advantages of protoxide of nitrogen as an anæsthetic in surgical operations—this, their final report, is to be found in the January and February Numbers of the *Missouri Dental Journal*—I will make a few quotations: February No., 1873, p.

42, says: "Independently of the above, the following objections may be urged against the view that nitrous oxide is decomposed in the blood. 1. The stability of the compound it not being decomposed by the ordinary deoxidizing agent. 2. The fact that when breathed, the quantity of the exhaled gases is much smaller than the quantity inhaled, which would not be the case were it decomposed in the lungs or blood vessels; for nitrous oxide being composed of two volumes of nitrogen, combined with one volume of oxygen, but occupying only the space of two volumes: any obstruction of oxygen would not be attended with any diminution of bulk. 3. If nitrous oxide be decomposed in the blood, in what manner can the liberated nitrogen, which must assume the gaseous form,—none, I think will venture to dispute—effect its escape through the walls of the blood vessels of the lungs? Yet its presence in the blood in the form of a gas, would we know, rapidly prove fatal to the individual.

I am, &c.,

A. COLEMAN.

"From these experiments so scientifically and carefully conducted, the majority of your committee are of opinion with Dr. Frankland and Mr. Coleman, that this agent can not produce its anæsthetic effect from being decomposed in the lungs or blood, as has been supposed by some; but are inclined to believe that it produces its effect by preventing oxidation of the blood, an opinion receiving support from the fact that anæsthesia may be produced by the inhalation of such gases as hydrogen, nitrogen, carburetted gases, &c."

From the above experiments we have the hitherto most satisfactory evidence and proof of the permanency of the gas, during its perigrinations through the circulation of the blood, at least for a short time, though decomposition may, and undoubtedly does after some time has elapsed, take place to a certain limited extent, otherwise animals immersed into the gas would sooner die. My own experiments with fresh meat, would favor this conclusion, also with mice.

Carpenter, in his Human Physiology, p. 313, edition 1865, says: "When nitrogen or hydrogen is breathed for any length of time, death results from the deprivation of oxygen, rather than from any deleterious influences which these gases themselves exert." On same page, speaking of the respiration of pure oxygen, says: "At first the rapidity of the pulse and the number of respirations are increased, and the animal appears to suffer little or no inconvenience for an hour; but symptoms of coma then gradually develop themselves, and death ensues in ten or twelve hours. If the animals be removed into the air before the insensibility is complete, they quickly recover. When the body is examined, the heart is seen beating strongly, while the diaphragm is motionless; the whole blood in the veins, as well as in the arteries, is of a bright scarlet color, and the blood coagulates with remarkable rapidity."

From what Mr. Carpenter says, we may conclude at least that nitrous gas is not at once decomposed in the blood, and rapid oxidation following as a natural sequence. If so, this blueness so often witnessed, would not take place; in fact, the symptoms of nitrous gas and oxygen, when breathed, are exactly opposite in the chief physiological effects on the body, and the experiments as reported by the committee above given, are also confirmatory of these conclusions.

The effects of the gas seems to be a downward grade instead of an upward one—a letting down of vital energy, a decline of power. Mr. Richardson's chilling process is simply to arrest a retarded oxidation, thereby lessening function and pain, pain being a morbid or abnormal function, and not a physiological process proper, hence pathological. I must regard the effect of any anæsthetic as pathological, and not a physiological effect; one pathological effect preventing another, must be the gas motto, (like *similia similibus curantur*, only in an opposite direction.)

Rapid oxidation going on in the economy, would necessarily increase traumatic pain, as in case of lancing a boil, felon, or cutting into any inflamed part; where there is undue oxidation going on, the nerves suffer more or less from *dialysis* of the part where there is an undue oxidation taking place, inflammation sooner or later follows, then too much heat is developed and too little magnetism, hence a disturbance in the *thermo magnetic* nerves, or currents, resulting in pain at the point or points.

There has been a great deal said and written on the subject of the safety of using gas indiscriminately for minor operations, when there is no danger from the shock of the operation even apprehended, still some deaths have occurred, and more will occur of necessity if the use of the gas is persevered in by the dental profession so unanimously as now practiced.

We naturally conclude from what Mr. Carpenter says concerning the inhalation of oxygen gas, that from the rapid oxidation of blood and tissues, a necessarily rapid formation of carbonic acid, an intense blueness of skin would follow, which, however, is not the case; on the contrary a florid condition throughout prevails. This is quite different from the effects of nitrous gas, which produces a cadaverous blueness so often witnessed in sudden death, as drowning and other causes of strangulation, and other modes of death. This blueness so far as it goes, means cadaver or death, nothing short, a tendency to decomposition, and suspension of nutrition, a loss of balance in the upward and downward forces: why? Because there is an abnormal condition.

An argument so often made use of by the advocates of the safety of gas, is that it does not contain any foreign body, only atmospherical elements. This is true, though not a very valid argument, as nitric acid, and all the other compounds of these two gases contain simply atmospherical elements, whose vapors inhaled, would *scuttle* the air passages like a ladle of melted mettle poured down the wind pipe.

Why write or say anything against nitrous gas, or any other mischievous humbug. People gulp the gas, and willingly have their mouths and pockets robbed, at the same time say it is a good thing, and let their teeth decay *ad libitum*, in consequence of gas; who can stop it? Still the world wags on, as this is a progressive age. It is a pity '*Josiah*' hasn't the same monopoly on gas that he has on rubber, so as to be able to fight it out on that line, right or wrong. If the use of gas could be made the exception and not the rule, it would be quite a different thing altogether. The abuse is what I object to, not its legitimate and discriminating use, which is all well enough.

